# Knowledge-Informed Machine Learning for Cancer Diagnosis and Prognosis: A Review

**DOI:** 10.1109/tase.2024.3515839

**Published:** 2024-12-18

**Authors:** Lingchao Mao, Hairong Wang, Leland S. Hu, Nhan L. Tran, Peter D. Canoll, Kristin R. Swanson, Jing Li

**Affiliations:** H. Milton Stewart School of Industrial and Systems Engineering, Georgia Institute of Technology, Atlanta, GA 30332 USA; H. Milton Stewart School of Industrial and Systems Engineering, Georgia Institute of Technology, Atlanta, GA 30332 USA; Department of Radiology and the Mathematical Neuro-Oncology Laboratory, Department of Neurosurgery, Mayo Clinic Arizona, Phoenix, AZ 85054 USA; Department of Cancer Biology, the Department of Neurosurgery, and the Department of Radiation Oncology, Mayo Clinic Arizona, Phoenix, AZ 85054 USA; Department of Pathology and Cell Biology, Columbia University Medical Center, New York, NY 10032 USA; Mathematical Neuro-Oncology Laboratory, Department of Neurosurgery, Mayo Clinic Arizona, Phoenix, AZ 85054 USA; H. Milton Stewart School of Industrial and Systems Engineering, Georgia Institute of Technology, Atlanta, GA 30332 USA

**Keywords:** Machine learning, deep learning, healthcare automation, cancer diagnosis, prognosis

## Abstract

Cancer remains one of the most challenging diseases to treat in the medical field. Machine learning (ML) has enabled in-depth analysis of complex patterns from large, diverse datasets, greatly facilitating “healthcare automation” in cancer diagnosis and prognosis. Despite these advancements, ML models face challenges stemming from limited labeled sample sizes, the intricate interplay of high-dimensionality data types, the inherent heterogeneity observed among patients and within tumors, and concerns about interpretability and consistency with existing biomedical knowledge. One approach to address these challenges is to integrate biomedical knowledge into data-driven models, which has proven potential to improve the accuracy, robustness, and interpretability of model results. Here, we review the state-of-the-art ML studies that leverage the fusion of biomedical knowledge and data, termed knowledge-informed machine learning (KIML), to advance cancer diagnosis and prognosis. We provide an overview of diverse forms of knowledge representation and current strategies of knowledge integration into machine learning pipelines with concrete examples. We conclude the review article by discussing future directions aimed at leveraging KIML to advance cancer research and healthcare automation. A live summary of the review is hosted at https://lingchm.github.io/kinformed-machine-learning-cancer/ offering an evolving resource to support research in this field.

## Note to Practitioners—

Recognizing the challenges posed by inter-patient and intratumoral heterogeneity, constrained sample size, and interpretability requirements in cancer applications, practitioners should consider integration of existing biomedical knowledge into their modeling frameworks. This strategy holds promise for enhancing model performance, robustness, and interpretability. We review classic machine learning and deep learning models that incorporated domain knowledge in their cancer diagnosis and prognosis models spanning models that use clinical, imaging, molecular, and treatment data. Pros and cons of each integration approach are discussed. Key design questions such as which knowledge to leverage, how to represent it effectively, and how to seamlessly integrate it into their models need be examined for each case. Collaboration between modeling scientists and medical experts is essential in this endeavor.

## I. Introduction

CANCER stands as a predominant cause of human death worldwide, with its incidence escalating alongside the increasing global life expectancy [1]. Despite its widespread occurrence, cancer remains among the most challenging issues in medicine due to the intricate genetic and pathogenic mechanisms underlying tumors. In recent decades, machine learning (ML) has positioned itself as a promising tool for analyzing complex patterns from large datasets, greatly facilitating “healthcare automation” in cancer diagnosis and prognosis. The computational power and versatility of ML has enabled automation for various tasks such as medical image segmentation, analysis of vast arrays of digital histopathology slides, interpretation of complex genetic profiles, and others.

Cancer applications present several significant challenges for the development of ML methods. The limited efficacy of conventional therapies is largely attributed to the pronounced heterogeneity inherent in tumors [2], [3], [4], [5]. At the individual-level, no two patients behave clinically the same, suggesting that conventional one-model-fits-all approaches are not sufficient. At the tumor level, genomics studies reveal spatial heterogeneity within tumors [6], [7], [8], [9], indicating the presence of distinct cellular subpopulations with varying phenotypic features even within the same sample of a tumor, a phenomenon known as *intratumoral heterogeneity*. Additionally, treatments such as chemotherapy exert selective pressure on tumor cells, leading to the dominance of treatment-resistant cells and driving disease progression, thereby reshaping the tumor microenvironment [2]. Given these intricacies, there is a pressing need to develop precise and effective ML models capable of characterizing the spatial landscape within tumors to facilitate targeted cancer therapies.

The effectiveness of ML models relies heavily on access to abundant and high-quality data. However, the process of labeling data often demands meticulous examination by medical professionals on a case-by-case basis. In reality, obtaining large quantities of tumor specimens for histopathological assessment is challenging, as only limited biopsies can typically be procured from specific tumor sites. These constraints pose obstacles to ML models in comprehensively learning the spatial landscape of tumors. Nevertheless, it is crucial to address the wide-ranging interpatient and intratumoral variabilities within and across tumors, as accurate predictions are essential for informing diagnoses and predicting and monitoring treatment responses for individual patients.

An additional significant challenge in ML for cancer applications lies in effectively analyzing diverse, multimodal, and high-dimensional datasets. Recent studies highlight the potential of combining individual genomic phenotypes with medical images, termed *radiogenomics*, as a promising alternative to invasive diagnosis [10]. However, the number of patient samples typically remains limited to hundreds, while the human genome encompasses tens of thousands of genes. ML grapples with the task of identifying relevant patterns amidst a vast array of genetic features intertwined with measurement noise, irrelevant genes, and inherent biological variability [11]. Developing models capable of efficiently integrating multimodal and high-dimensional data within the constraints of small sample sizes is an important direction for enhancing AI-assisted cancer prognosis.

Lastly, the lack of interpretability in ML models can erode trust in their role as decision-support tools and hinder the clinical utility of the predictions. Deep learning (DL) models, in particular, often face criticism for being “blackbox machines,” with mechanisms that are unintelligible and unverifiable to humans. To address this concern, Explainable AI (XAI) techniques have emerged, seeking to quantify the importance of features in the model outputs [12]. While these interpretation tools enable practitioners to validate model behavior against medical knowledge, they are typically applied retrospectively. A more proactive approach involves integrating the desired behavior into the model training process.

To tackle the aforementioned challenges, a promising approach involves incorporating biomedical knowledge into ML models, a method known as *knowledge-informed machine learning* (KIML). By integrating domain knowledge (or models of that knowledge) into the learning process, the accuracy, robustness, and interpretability of models can be improved. This strategy has gained traction over the past decade, proving effective in various scientific, engineering, and healthcare contexts, especially in scenarios with constrained training data.

Several reviews have explored concepts related to KIML. A pioneering study [13] established the concept of theory-guided data science, detailing methods for integrating scientific knowledge—such as physical constraints, scientific principles, and theory-based models—with data-driven models. Following this, research in the biological, biomedical, and behavioral sciences outlined strategies for combining ordinary and partial differential equations with ML [14]. Another study examined methods for incorporating prior knowledge into DL models within the context of dynamical systems [15]. More recently, von Rueden et al. developed a comprehensive taxonomy of KIML [16], categorizing it into three dimensions: the source of knowledge, its representation, and its integration into the machine learning pipeline. While this review provided an extensive overview of concepts, it primarily focused on general methodologies, with limited emphasis on the medical and healthcare fields—domains characterized by rich domain knowledge, limited labeled samples, and a pressing need for improved model generalizability and interpretability. Considering the significant clinical challenges associated with cancer treatment and the promising potential of ML to advance healthcare automation, we provide a comprehensive review of KIML applications in the cancer domain.

**Fig. 1.**
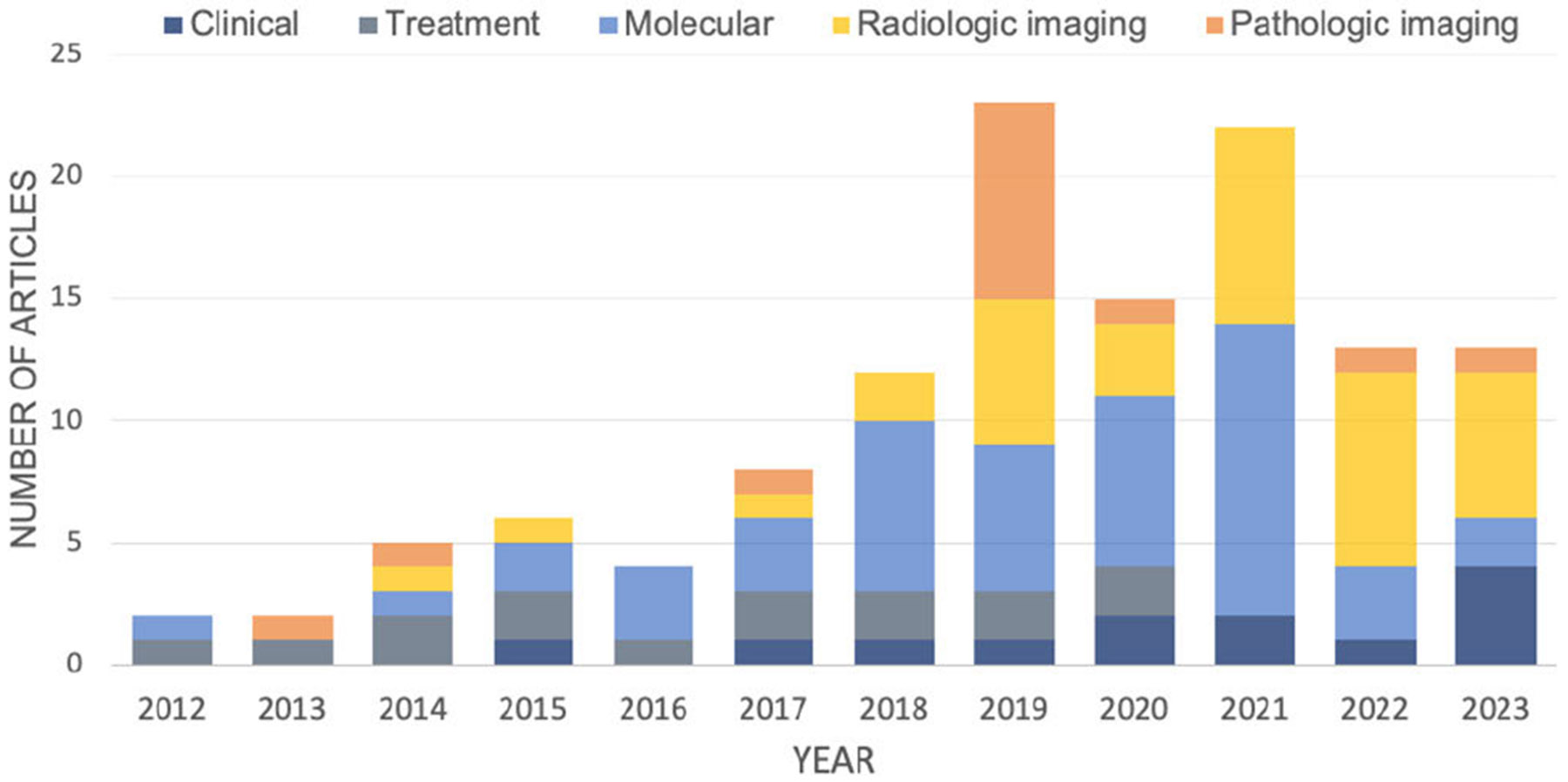
Annual trends of knowledge-informed machine learning articles addressing cancer diagnosis and prognosis.

The objective of this paper is to present a systematic review of KIML techniques for cancer diagnosis and prognosis. The scope encompasses both classic ML and DL models employing input data such as gene expression profiles or medical images to predict clinical outcomes such as cancer phenotype or patient risk score. An extensive search was conducted on PubMed in April 2023, focusing on original research articles published from 2012 onward (within the last 10 years). The search was employed via the concatenation of three keywords: (*knowledge-informed, physics-informed, knowledge, informed, theory-guided, mathematical model)* and *(machine learning, deep learning)* and *cancer*. We acknowledge that this search criteria might not cover all important work in the field. A total of 125 articles met the inclusion criteria for this review (Figure 1). We host a live summary of this review at https://lingchm.github.io/kinformed-machine-learning-cancer/, which will be updated periodically, providing an evolving resource for the community.

**Fig. 2.**
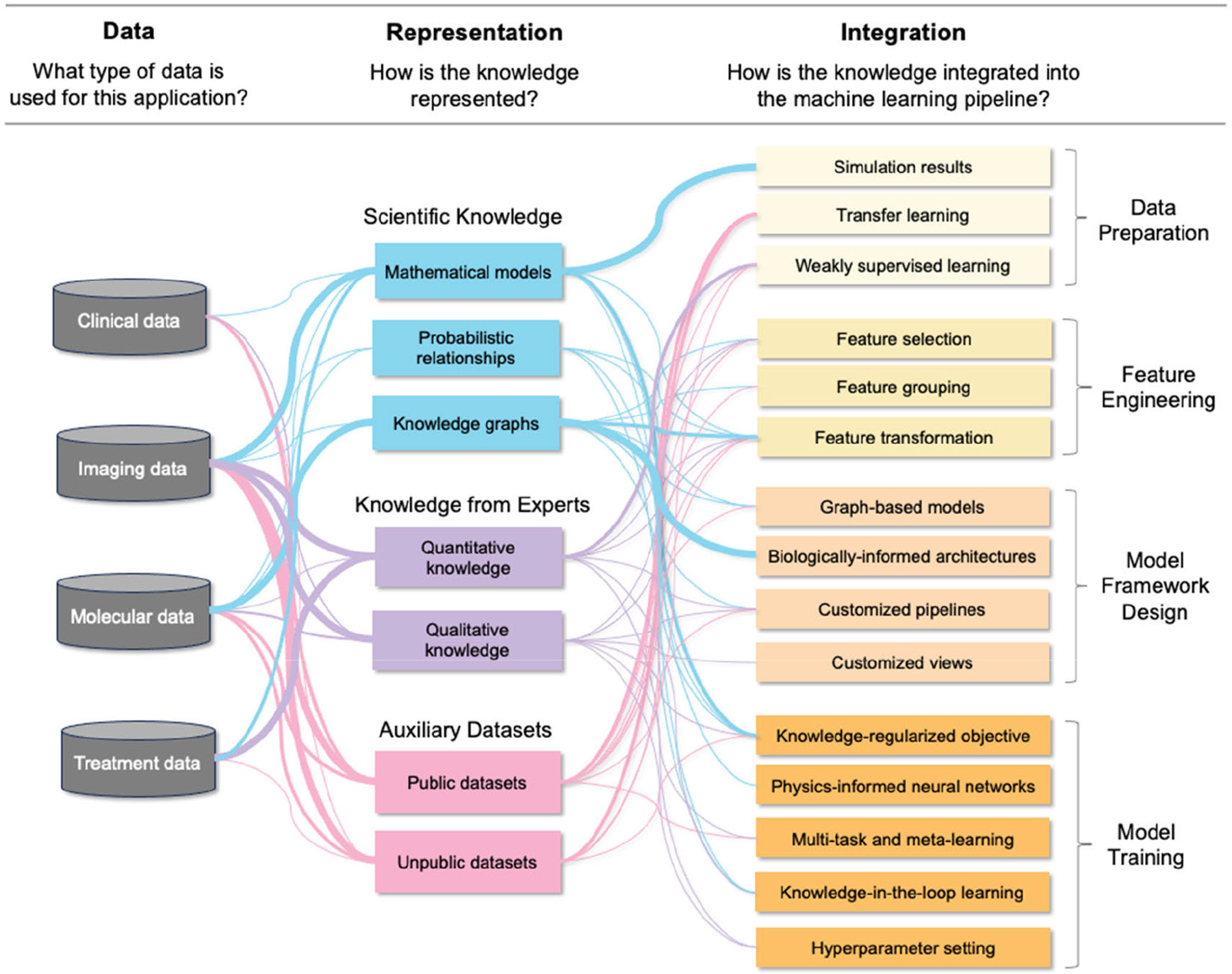
Taxonomy of knowledge-informed machine learning in cancer diagnosis and prognosis.

In our review, we structure our analysis around three key components, as illustrated in Figure 2. Firstly, we examine the four primary types of data essential for cancer diagnosis and prognosis. Next, we explore various methods of representing biomedical knowledge. Subsequently, we offer a detailed examination of mainstream approaches for integrating knowledge across different stages of the ML process, encompassing data preparation, feature engineering, framework design, and model training. Finally, we discuss prospective avenues for further developing KIML to enhance the accuracy of cancer diagnosis, prognosis, and treatment response prediction.

## II. Types of Input Data

A foundational review on clinical decision-support systems (CDSSs) defined four types of features that should be considered when developing a prediction model [17]. Adhering to this framework, we describe four primary types of data in cancer diagnosis and prognosis: clinical, imaging, molecular, and treatment data. An overview of their capabilities and limitations is provided in Table I.

### A. Clinical Data

Clinical data encompass a spectrum of patient-related information, spanning demographics, medical history, laboratory test results, symptom logs, performance status, questionnaires, health records, and more. Typically, clinical data can be obtained with relative ease [17], though the use of validated scoring systems is imperative for ensuring standardized clinical measures across medical centers. A large portion of clinical data is provided in text form, such as electronic health records (EHR), nursing notes, pathology reports, radiology reports, and more. These diverse data sources offer diverse perspectives on the patients’ health condition.

With the emergence of language models, there is a growing research trend to employ DL techniques for the automatic annotation of clinical notes with disease outcomes such as cancer metastasis, disease stage, or tumor recurrence. In particular, Large Language Models such as BERT [18], BioBERT [19], BioMegatron [20], and clinicalBERT [21] have demonstrated promising potential for automated knowledge extraction from textual data. Pre-trained on large biomedical text corpora such as PubMed, these models can support clinicians in the evaluation of their findings in comparison to existing evidence in the literature [22]. Text data is typically analyzed separately from other data modalities and using different model architectures due to inherent differences in data format and information density. The fusion of unstructured and structured data modalities is an active area of research.

### B. Imaging Data

#### 1) Radiologic Imaging:

Radiologic imaging constitutes a routine component in the clinical diagnosis, surveillance, and treatment monitoring of tumors. Common types of radiologic imaging employed in cancer applications include Magnetic Resonance Imaging (MRI), Positron Emission Tomography (PET), Computer Tomographic (CT), X-rays, ultrasound, and mammography. Radiologic imaging allows capturing physiological and morphological characteristics of the tumor in a noninvasive manner. Despite these advantages, radiologic images can be limited by the spatial resolution and noise of the imaging technology. The presence of artifacts stemming from factors such as movement, scatter, attenuation, and partial volume effects (PVEs) can further impact imaging quality, potentially leading to ambiguous tumor boundaries [23].

In the clinical domain, the diagnosis of suspicious lesions as benign or malignant rely on the visual interpretation and expertise of radiologists, introducing potential inter-observer variability [24], [25]. Computer-aided diagnosis systems allow for more reproducible descriptors through systematic processing of quantitative tumor features [26]. Recently, ML has gained widespread application in analyzing radiologic images, facilitating automated tumor segmentation, diagnosis, and monitoring. These AI-powered systems hold promising potential for expediting the time-consuming human examination process. A prevalent approach involves building whole-tumor classification models, generating a singular predicted outcome for each tumor [27]. However, given the intratumoral heterogeneity, a spatially resolved assessment of tumor landscape is needed to provide better therapeutic value. Radiomics models capable of delivering predictions across each spatial unit of the tumor can be used to identify tumor regions with genomics alterations [28].

Another pertinent research question is how to integrate multimodal images with missing data. Consider multiparametric MRI as an example. Each imaging sequence unveils distinct characteristics of the tissue: T1-weighted sequences reflect blood-brain barrier and regional angiogenesis; T2-weighted sequences can be used to assess extracellular fluid in brain parenchyma; and Diffusion tensor imaging (DTI) aid determining the water diffusion process, which is affected by tumor cell architecture and density [29]. Multimodal imaging data affords a more comprehensive understanding of the tumor and surrounding abnormal tissues [30], [31], however, collection of multimodal data for every patient may not be feasible due to economic or practical constraints. How to leverage incomplete-modality data and maintain prediction performance for patients who only possess a subset of modalities is an active area of research.

#### 2) Pathologic Imaging:

In contrast to radiologic images that provide high-level characterization of the tumor, pathologic images focus on sparsely sampled cell-level evaluations for each localized tissue. While radiology has a long history of research and clinical application, pathology just underwent a digital revolution after Food and Drug Administration’s approval of digitized whole-slide images in 2017 [32]. With enhanced storage and data management capabilities, digital pathology slides have proliferated as an indispensable instrument in clinical diagnostics, drug development, tissue-centric research, and other scientific endeavors.

The relative novelty of digital pathology and high cost of manual microscopic evaluation are reasons for the lack of large, annotated datasets. As pathologists are trained to follow algorithmic decision trees to assess the specimens, notable *inter-observer variability* exist [33]. A retrospective study [34] found that pathology review by experts changed the nodal status in 24% of patients. Another challenge is the intrinsic high-resolution of whole-side images (WSI) rendering them too large to accommodate under memory and computational constraints. A prevailing approach is to partition WSIs into patches for model training, then aggregate patch-level predictions into slide-level diagnosis. However, the patch-based approach entails a compromise on the broader visual context, potentially resulting in an incomplete representation of information crucial for diagnosing specific phenotypes [35]. An additional stratum of challenge arises from the frequent unavailability of region-specific annotations that precisely identify tumor indicators, instead of image-level annotations, owing to the time-intensive labeling process. There is no recognized best strategy for how to map image-level labels to patch-level partitions and how to aggregate patch-level results into image-level diagnosis.

### C. Molecular Data

Cancer is essentially a disease of the genome which evolves with accumulations of somatic mutations. Genomics data plays a pivotal role in disease diagnosis, drug development, and the identification of biomarkers and immune signatures. Tailoring molecularly targeted treatments based on genetic changes offers a personalized approach to enhance clinical outcomes beyond conventional one-size-fits-all treatments.

Advances in gene sequencing and computational tools have enabled comprehensive study of the entire genome, encompassing genomic, epigenomic, proteomic, transcriptomic, metabolomics, and other -omic levels [36]. In essence, *molecular omics data* characterize the abundance of molecules in tissue samples, providing intricate insights into cancer clonal evolutions, tumor heterogeneities, and cell radiosensitivity at a much higher resolution [17]. Another type is *molecular interaction data*, which describe the potential function of molecules through interacting with others [36], such as protein-protein interactions and protein-RNA interactions. Various molecular interaction datasets, derived from large-scale cell line studies, are publicly available [37], [38], [39], [40], [41], [42], [43], [44].

A benchmarking review of drug sensitivity prediction models found that models using multiple omics profiles exhibited the best performance for drug response prediction in breast cancer cell lines, suggesting that different omics profiles provide complementary information for the predictive task [45]. The authors also emphasized the importance of incorporating prior biological knowledge regarding breast cancer oncogenes and disease-driving pathways [45].

Despite the inherent strengths of molecular measurements, the acquisition of tumor specimens is inevitably expensive. Obtaining biopsy samples necessitates surgery and subsequent preprocessing for sequencing tests, and due to practical constraints, only a limited number of biopsy samples can be collected from restricted locations. This leaves a substantial portion of the tumor region unmeasured. The emergence of liquid biopsy, which collected through a peripheral blood draw, offers a less invasive alternative. However, not all clinically relevant biomarkers can be detected from liquid biopsy, and many tests derived from this venue require further clinical validation [46], [47]. Moreover, conducting molecular tests on every tumor is sometimes prohibitive, as treating a patient with multiple drugs to study the independent effects of each drug is impractical. A viable alternative is to conduct pre-clinical experiments using human cancer cell lines or animal models. Assuming that genetic associations remain consistent to some extent between cell line and patient tumor samples, larger pre-clinical datasets provide valuable insights for clinical models of drug response prediction [48], [49].

### D. Treatment Data

Conventional cancer therapies involve chemotherapy, surgery, and radiotherapy. Surgery that majorly removes tumor is most effective at early stages, however, the possibility of tumor recurrence is high [50]. While chemotherapy and radiotherapy can be used before and/or after surgery to eliminate the remaining tumor, tumor cells may become resistant to the chemotherapy drug [50]. Features extracted from planned spatial and temporal distribution of the radiotherapy or chemotherapy dose provide valuable information for predicting tumor recurrence and post-treatment progression [17]. Various studies have integrated treatment plans, dose reconstructions, or historical clinical drug doses as either predictive features or ground truth labels in ML models [51], [52], [53], [54], [55], [56], [57], [58], [59], [60], [61], [62], [63], [64]. Salient works utilized patient-specific anatomy features and planning parameters in their dose prediction models [51], [55], [63]. Typically, doses are reported in terms of mean dose applied to the entire tumor or doses to a prescription point inside the tumor, although recording spatially variable dose distributions can provide more value for models that consider intratumoral heterogeneity [65].

## III. Types of Knowledge Representation

The form in which knowledge is encoded shapes how it can be integrated into ML models. In this section, we describe diverse forms of knowledge representation. Table II provides an overview.

### A. Scientific Knowledge

Knowledge in this category is obtained through a series of observations, phenomena, formulation of hypotheses, and validation using scientific methods. In the following sections, we discuss three prevalent representations of scientific knowledge in KIML. It is important to note that some works extend beyond these three categories [27].

#### 1) Mathematical Models:

The growing use of mathematical models to integrate and test existing hypotheses and knowledge in cancer biology have driven forward the field of Mathematical Oncology over the last few decades [66]. The advantage of employing this form of knowledge lies in its well-defined nature and extensive validation through experiments like those using foundational mathematical biology approaches summarized in the pioneering book “Mathematical Biology”, leading to a diversity of insights across cancer applications [67], [68]. These mathematical models play a pivotal role in integrating and testing hypothesis in cancer research.

Several mathematical models have been established for cancer growth [69], [70], [71], immune system-tumor interactions [72], normal tissue complications in radiotherapy [73], imaging physics [74], [75], or thermal dynamics [76]. For instance, the proliferation-invasion (PI) [177] model adapted the classical convection–diffusion equation [68] to simulate the growth and invasion of tumor cells in glioblastomas (GBM):

(1) $$\overset{\begin{array}{c} rate\;of\;change\;of\\ tumor\;cell\;concentration \end{array}}{\overset{︷}{\frac{\partial c}{\partial t}}}=\overset{diffusion}{\overset{︷}{\nabla \cdot (D\nabla c)}}+\overset{proliferation}{\overset{︷}{\rho c\left(1-\frac{c}{K}\right)}},$$

where c is the concentration of tumor cells (cells/mm^3^), t is time, D is the net rate of diffusion (mm^2^/year), $\nabla^{2}$ is the dispersal operator, ρ is the net rate of proliferation (1/year), and K is the limiting concentration of cells that a volume of tissue can hold (cells/mm^3^). This model has been widely used to estimate intratumoral tumor cell density [175], [176], [177], radiation sensitivity [178], [179], treatment response [180], [181], [182], [183], and gene mutation status [69], [75], [175], [176], [177], [184].

Mathematical models and ML serve distinct yet complementary roles in understanding tumor-environment states. The former offers mechanistic understanding of tumor dynamics, while the latter explores data-driven patterns from supplied data. KIML models embrace the complementary strengths of these approaches [185], [186], [187]. For example, scalar parameters from mathematical models can be replaced with patient- and/or time- dependent variables estimated from patient-specific datasets using ML [80], [81], [82], [83], [84]. Conversely, mathematical models can be wrapped into the optimization objective of ML models converting them into knowledge-informed loss functions [76], [95], [96].

Nevertheless, certain mathematical models cannot be easily integrated into ML models due to their intricate formulations, such as those lacking closed-form solutions. To overcome this limitation, simulation proves to be a widely employed numerical method. Typically, a simulation engine employs numerical methods to solve a mathematical model and produces outcomes based on specific parameters within a given context. Example works that included this type of knowledge are a partial differential equation (PDE)-based simulator applied for brain tumor [86], [97] and a simulation-based kernel built for kernelized ML models applied for anti-cancer drug sensitivity prediction [98]. Calibration of mathematical models using parameter estimates obtained from experimental data is another venue to reduce the parameter space. For parameters that are difficult to obtain experimentally due to measurement challenges or unintelligible physics of the environment, ML has been a provable venue to estimate suitable parameter values through learning input-output mappings [69], [94], [99].

#### 2) Probabilistic Relations:

Knowledge in this context can be represented through correlation structures, probability distributions, or conditional independence relationships between random variables. Examples of integrating this type of knowledge into ML models include utilizing the differential dose-volume histograms (DVHs) distribution in predicting radiation therapy outcome [51], [100], incorporating gene mutation rates for different cancer types derived from previous studies to enhance cancer prognosis [101], utilizing a probability atlas containing prior probability of certain organs appearing at each pixel location to aid tumor segmentation [102], [103], [104], and leveraging zonal probabilities that indicate where aggressive cancer is more likely to occur as the prior in Bayesian models [105].

A representative example is the mathematical framework for DVHs predictions. DVH [106] is one of the most commonly used metrics in radiation oncology for evaluating the quality of an intensity modulated radiotherapy (IMRT) plan. The treatment target is divided into sub-volume $V(A_{jk})$. For each sub-volume, a differential DVH, $dV\left(A_{jk}\right)∕dD$, is calculated and fitted to a skew-normal probability density function,

(2) $$f(p_{1},p_{2},p_{3};D)=\frac{1}{\pi p_{2}}\;\text{exp}\left(-\frac{{\left(D-p_{1}\right)}^{2}}{2p_{2}^{2}}\right)\;\;\times \int_{-\infty }^{\frac{p_{3}(D-p_{1})}{p_{2}}}\text{exp}\left(-\frac{t^{2}}{2}\right)dt,$$

where $p_{1}$, $p_{2}$, $p_{3}$ are parameters of location, scale, and shape. Such knowledge have been incorporated in DL models to improve the quality of treatment plans [51], [100].

#### 3) Knowledge Graphs:

Relationships between gene expressions and higher-level biological entities can be naturally represented as knowledge graphs. Commonly used publicly available knowledge graphs include the Kyoto Encyclopedia of Genes and Genomes (KEGG) [37] database with interaction probability of pairs of genes and proteins, protein–protein interaction database (STRING) [39], molecular signatures database (MSigDB) [41], Gene Ontology [40], gene connection and function prediction (GeneMANIA) [44], and the Reactome [38] pathway database. For example, KEGG has been used to design KIML models to enhance model performance across various tasks such as prediction of survival of lymphoma and ovarian cancer patients [107], lung cancer patients [108], [109], and GBM patients [110], diagnosis of the grade of Low-Grade Glioma and GBM tumor samples [111], and prognosis of metastasis for breast cancer patients [112].

### B. Expert Knowledge

Expert knowledge, in our framework, is defined as knowledge held within a specific group of experts and cultivated within their community. Certain aspects of this expertise are extensively studied and documented, while other facets are directly derived from domain experts, implicitly validated through their cumulative experiences.

#### 1) Quantitative Expert Knowledge:

Quantitative knowledge refers to information that employs numbers, measurements, statistics, logical relationships, and other quantitative methods to articulate connections between distinct variables within a system or process. This category of knowledge is typically well-defined and mathematically expressed, making their integration into ML models convenient. While these type of feedback from experts is typically directly related to the target task, their acquisition require time-consuming assessments.

A primary category of quantitative knowledge from experts encompasses diagnoses and annotations. Several works have developed knowledge-based treatment planning models by leveraging physician satisfied treatment plans or clinical plan doses for prostate cancer [51], [53], [55], [56], [59], [63], [113], head and neck cancer [54], [60], [62], [63], [64] and oropharyngeal cancer [57], [58]. A representative example by Chanyavanich et al. [53] assembled a database of a hundred prostate cancer IMRT treatment plans, all reviewed and delivered by experts. Each new case was matched to a prior reference case within the knowledge based on the relative spatial locations of target volume and normal structures. The treatment plan parameters from the matched case were used as an enhanced starting point in the planning process. Similarly, to diagnose a common adverse effects of chimeric antigen receptor therapies in cancer treatment, Bogatu et al. [114] defined a knowledge base (KB) of statistical biomedical facts extracted from domain literature. The KB was used to augment samples of biomarker concentration measurements through quantifying the degree of similarity between the given measurement value and the statistical values reported in each study in the domain literature [114]. Beyond the diagnosis, experts’ estimations of disease progression offer valuable supplementary information. In a detection and diagnosis model of lung cancer, malignancy scores estimated by radiologists were employed in model training as imprecise labels [115]. Further, coarse annotations or drawings of abnormal regions by pathologists have been utilized to aid cancer type classification and image segmentation [116], [117], [118].

Experts may provide knowledge in the form of ordering relationships or correlations between entities. A paradigm of using ordinal relationships is provided by Mao et al. [119] in brain tumor prognosis, where ordinal relationship concerning tumor cell density in samples from different areas were used to extract weakly labeled samples. A similar strategy was adopted to model knowledge-determined ordinal relationships between labels for genetic status prediction [120]. Both methods significantly outperformed traditional models that did not incorporate ordinal relationships, showcasing their effectiveness in handling inter-patient and inter-tumor variability as well as small labeled sample sizes. On the other hand, Hu et al. [121] observed a consistent positive correlation between relative cerebral blood volume (rCBV) and tumor cell density and leveraged this information to constrain knowledge transfer from other patients.

Another type of quantitative knowledge pertains to estimated sizes or shapes of lesion areas. For example, Zhou et al. [122] noted that tumors generally exhibit the largest aspect ratio in the coronal view, and the shape of tumors in the sagittal view is closer to square than in the other two views. Leveraging this information, appropriate anchor sizes and aspect ratios for each view were specified, forming the basis for constructing candidate bounding boxes for view-specific tumor detection models. A similar example of using expected size and shape distributions of nodules in a DL segmentation model was done by Liu et al. [123].

#### 2) Qualitative Expert Knowledge:

Qualitative knowledge encompasses information derived from observations, interviews, subjective experiences, and behaviors of experts from their clinical practice. While this form of knowledge is typically available at low acquisition costs, they are not well-defined and are accompanied with subjectivity. Adapting the model behavior to follow such “expert experience” require more customization efforts.

Certain qualitative knowledge about diseases or human biology, while seemingly intuitive to humans, can significantly enhance the accuracy and interpretability of ML models. For instance, tumors of the same category are often found in similar anatomical locations: gliomas typically involve white matter, meningiomas are commonly adjacent to the skull, gray matter, and cerebrospinal fluid, and pituitary tumors are often located near the sphenoidal sinus, internal carotid arteries, and optic chiasma [29]. Chen et al. [124] proposed a tumor region augmentation and partition strategy to take into consideration the differently weighted informative context surrounding the tumor. Other qualitative aspects such as the smoothness of morphological characteristics of cells within a WSI [125], sparsity of predictive gene expressions [126], transferability of drug sensitivity between cell lines and patient tumor samples [48], boundary ambiguities of the lesion in MRI [51], experts’ validation and feedback [127], [128], and visual heterogeneities [130], [144] have been attempted to account for in KIML.

Another qualitative knowledge shared by the medical community are domain-specific lexicons. Liu et al. [130], [131] curated a lexicon comprising relevant clinical words and lists of synonyms extracted from a small sample of radiology reports. This lexicon was used to standardize and trim down input tokens into clinically relevant vocabulary. Guan et al. [132], on the other hand, employed the National Library of Medicine [133] to find gene terms associated with lung cancer.

Experts may also serve as behavioral references for ML models [35], [134], [135]. Liu et al. [123] captured sonographic characteristics that radiologists focus on when examining ultrasound images. The datasets were then categorized into multiple groups based on the sonographic characteristics selected by radiologists. Similarly, Corredor et al. [135] tracked the gaze of pathologists when they used a navigation window to examine WSI images of the skin. A likelihood of cancerous was assigned to each nucleus based on the number of times the region was examined by pathologists. Drawing inspiration from actual clinical decision-making processes, models have been designed to mimic various zoom levels [136] and different visual cues [137] that pathologists use in their diagnosis.

### C. Auxiliary Datasets

Utilizing an auxiliary dataset can yield several advantages, including: (i) *Improved generalizability*: the inclusion of data from external sources can broaden the distribution encountered during training, enhancing the model’s ability to learn more robust relationships within the data; (ii) *Domain adaptation*: an auxiliary dataset can facilitate the adaptation of a pre-trained model to a new domain or task. This transferability can alleviate the sample size needs of data-hungry DL models.

#### 1) Public Datasets:

To encourage collaboration in cancer research, there is increasing research efforts to compile and release the data collected from multiple large-scale preclinical and clinical studies, such as the National Cancer Institute’s Cancer Research Data Commons [138], the Cancer Cell Line Encyclopedia [139], the Cancer Genome Atlas (TCGA) [140], International Cancer Genome Consortium [141], Genomics of Drug Sensitivity in Cancer [142], and the Cancer Dependency Map (DepMap) [143]. These public datasets typically encompass large patient cohorts with different cancer types and conditions to serve diverse research topics and applications. The availability of public datasets has greatly fostered collaboration, transparency, benchmarking in ML research. Researchers need to be aware of the data quality variability and differences in data acquisition protocols, equipment, batch effects when using public datasets.

Several studies have demonstrated that harnessing large public datasets, even those unrelated to the cancer domain, can enhance model performance on cancer applications. For instance, Vu et al. [144] and Wang et al. [145] utilized ImageNet for pretraining tumor segmentation and tumor classification models. Liu et al. [130] employed the BERT model pretrained on millions of words from the internet as well as Wikipedia articles to augment training data for their cancer classification model from radiology reports. Another interesting use case by Kwon at al. [146] leveraged healthy tissues from the Cancer Imaging Archive to improve generalizability of their tumor segmentation model.

#### 2) Unpublic Datasets:

In most research projects, data is typically gathered and owned by specific organizations with access restricted to authorized individuals or entities. These datasets are common particularly in healthcare, where protecting sensitive patient information and compliance with legal and ethical standards is paramount. This exclusive access allows researchers to have a better control over data quality and tailor the data collection to specific research questions. Representative use cases of these auxiliary datasets include: inclusion of other imaging sequences from the same patient [147], using data from other patients with the same type of disease [135], [145], [166], [167], [168], examination of patients with different types of cancer or drugs [109], [152], formulating different tasks within the same domain [153], transfer knowledge learnt from pre-clinical data [49], using a comprehensive database that contains progression notes, radiology reports, nursing notes [154], or leveraging a collection of physician-approved treatment plans [53], [155].

In addition to labeled datasets, there may exist unlabeled or baseline datasets from which the model can extract knowledge relevant to the predictive task, such as unlabeled specimens from other patients [156], [157] or data from healthy controls [149], [158]. An representative example of this approach first extracted a lower-dimensional representation of normal brain appearance using T1 MRI data of healthy controls [149]. Images of patients with brain tumors were then projected into this representation to obtain a reconstruction of their “normal brain.” The feature maps of tumor patients and healthy controls were then aligned using a Siamese network, and regions with low feature consistency between the feature maps of normal and tumor images were segmented as tumor regions.

## IV. Type of Knowledge Integration

In this section, we review how biomedical knowledge can be integrated into ML models. We categorize knowledge integration strategies according to which stage of the ML pipeline the integration happens: data preparation, feature engineering, framework design, and model training. How to integrate knowledge depends on the type of knowledge that is available. Table II summarizes integration strategies that have been applied to different forms of knowledge representation.

### A. Data Preparation

Augmenting training data with additional datasets generated from prior knowledge is a versatile approach in KIML. Techniques such as transfer learning and weakly supervised learning can effectively combine original data with knowledge-generated data for model training.

#### 1) Simulation Data:

Synthetic data generated through simulation can be combined with observed data for model training [23], [50], [71], [72], [73], [74], [75], [76], [77]. A representative example is the widely employed computer-based glioma image segmentation and registration (GLISTR) algorithm [146]. GLISTR takes a generative approach to produce patient-specific tumor segmentation maps. The process initiates with a set of normal atlases derived from the healthy population, each defined as a probability map for white matter, gray matter, and cerebrospinal fluid. The atlases are then customized to patient-specific maps by simulating tumor growth using the diffusion-reaction-advection model.

Simulated images can effectively pre-train DL models. In a recent study on tumor segmentation from PET scans [23], first- and second-order tumor descriptors were used to generate simulated images that replicated the on the physics of PET. These simulations included a range of realistic background intensities from various patients to emulate patient heterogeneity. The DL model was pre-trained on a large set of these simulated images and then fine-tuned with a smaller collection of real PET images. This approach demonstrated significantly better predictive accuracy compared to other methods, including semi-automated segmentation, thresholding-based techniques, active contour methods, and clustering approaches. Moreover, the model exhibited enhanced generalizability across data from five different scanners and performed well even with limited training images.

#### 2) Transfer Learning:

Transfer learning (TL) stands out as a widely employed technique for knowledge integration [22], [49], [98], [111], [135], [141], [152], [153], [154], [155], [156], [162], [165], [166], [203], [204]. It is assumed that by pre-training on an extensive and diverse dataset, the model acquires generally useful feature representations and thus only requires minor fine-tuning on a small dataset to perform the target task. TL proves particularly valuable in scenarios where there is limited labeled data for the target task, and a substantial amount of labeled data is accessible for other related tasks or from a similar domain.

TL enables flexible transfer of knowledge learnt in different domains. Knowledge can be transferred across patients with the same disease, where a model trained on a comprehensive dataset of historical patients captures population-level patterns, and a smaller, patient-specific dataset is used to bias the model for each individual [119], [121], [151], [157]. Similarly, knowledge can be transferred across different diseases. López-García et al. [109] conducted pre-training of a CNN model using gene expression profiles encompassing over 30 cancer types and fine-tuned the model on data specific to lung cancer patients. Knowledge can even be transferred across different data modalities. For example, a model trained on more accessible imaging modalities (e.g., T1-Gd MRI) can be transferred to modalities with less imaging data [102], [150]. This has proven benefits in medical imaging applications where patients may undergo varying numbers of imaging exams based on factors like physician preference, cost considerations, and center availability. Moreover, models trained for different ML tasks (e.g., tumor detection, tumor classification, tumor segmentation) using the same dataset can leverage shared learned feature representations [153].

Large scale pre-trained language models, including BERT [18], have gained popularity in various domains. Biomedical variations of BERT, such as BioBERT [19] and BioMegatron [20] trained on biomedical research articles, RadBERT [166] trained on radiology reports, and ClinicalBERT [21] on clinical notes, have been developed. These pre-trained models are later fine-tuned using in-domain data, such as electronic health records from a small group of patients [130], [163]. Some studies [144], [145] also demonstrated value of pre-training on non-medical images. While pre-training is typically seen in studies that use public large-scale datasets [111], [141], [152], [153], [154], [155], other types of TL such as instance transfer and feature transfer are more used for unpublic datasets [135], [165], [166], [167].

The extent of knowledge transferred can be adaptive, contingent on the knowledge- or data-driven similarity between domains or patients. An effective TL algorithm should determine which knowledge, and from which source domains or patients, is transferrable to the target domain or patient. A case of controlled knowledge transfer is exemplified by Chanyavanich et al. where the authors compiled a database of physician-approved treatment plans and implemented an algorithm to identify analogous patient cases from the database [53]. Once the optimal match was identified, the clinically approved plan was incorporated to formulate a treatment plan for the new patient. In another example by Hu et al. [121], knowledge transfer was constrained to patients exhibiting a substantial positive threshold between the rCBV measures from contrast MRI and tumor cell density to avoid negative transfer.

#### 3) Weakly Supervised Learning:

In addition to TL, weakly supervised learning (WSL) serves as another approach to augment training data and incorporate knowledge [32], [128], [130], [131], [132], [144], [157], [158], [191], [202], [204], [206]. Weakly supervised models can accommodate samples with imprecise or incomplete labels, particularly suited for scenarios where obtaining fully labeled data is costly or practically challenging.

*Imprecise* labels provide inexact or coarse-grained information about the ground truth. This scenario is common in pathological applications involving WSI. As discussed earlier, WSI slides are often divided into smaller patches for model training, yet only slide-level annotations are available. Imprecise labels indicate that if a slide is positive, at least one patch must contain a tumor, and if a slide is negative, then all tiles must be devoid of tumors. Multi-Instance Learning (MIL) is a prevalent approach to address such challenges and have and has demonstrated promising results across various cancer types, including prostate, skin, and breast cancers [32]. The MIL formulation induces learning of a patch-level representation that can separate the discriminative patches in positive slides from all other patches. Another strategy is selfdistillation, which separates weakly labeled data into high and low fidelity samples, selecting only the most representative high-fidelity data for noise-robust training [171]. Alternatively, weak labels can be constructed from radiology reports [169] or supplemental clinical data sources [172].

*Incomplete* supervision occurs when only a limited subset of training samples is labeled, leaving the remaining samples unlabeled. Integrating unlabeled samples into the training process can broaden the feature space and enhance the diversity of instances, thereby improving the robustness of the model. Semi-supervised learning strategies are often employed in such scenarios. Even though specific label measurements are absent for each sample, higher-level domain knowledge may exist regarding the unlabeled samples. For instance, Wang et al. [164] utilized the hierarchical relationship among three tissue-specific gene modules —proliferating tumor cells, reactive/inflammatory cells, and infiltrated brain tissue— to predict regional distributions of intratumoral heterogeneity. By integrating implicit biological knowledge with large volumes of unlabeled data, their model achieved significantly better predictive accuracy compared to various deep learning and traditional machine learning approaches. Another form of incomplete supervision leverages ordinal relationships among unlabeled samples [119], [129], [170]. Wang [170] proposed a classifier that could leverage interval labels (samples belonging to either class 1 or 2) when predicting tumor cell density using neuroimaging. A mathematical constraint was imposed to guide the model not to classify these samples into class 3, even though their belonging to whether class 1 or 2 is unknown. This KIML approach can be flexibly used in other datasets and applications where ordinal relationships among classes is partially known.

### B. Feature Engineering

Feature engineering is a critical part of ML involving the transformation of raw input data into a format suitable and effective for model training. A comprehensive simulation study of phenotype classification and drug response prediction showed that incorporating biological knowledge about either predictive genes or signals downstream in a pathway can be more useful than providing the complete set of genes and would require significantly less training data than uninformed learning [173]. Thus, domain knowledge can play an important role in feature engineering.

#### 1) Feature Selection:

In applications involving textual data, standardized medical language databases such as the Unified Medical Language System (UMLS) [174] and Metathesaurus [175] can be used to curate lexicons of clinically relevant terms and their synonyms. When dealing with gene expression data, a common strategy is to select features based on gene sets or pathways known to be associated with the disease of interest [52], [132], [176]. However, focusing only on known biological knowledge may restrict the exploration of gene-disease relationships that are yet undiscovered. Additionally, the disease marker identification may not fully account for higher-order interactions of genes related to the disease.

A more adaptable strategy involves combining data-driven and knowledge-informed feature selection. A comparative study of 14 published methods for patient risk stratification in breast cancer indicated that incorporating prior knowledge into ML models can enhance gene selection stability and biological interpretability [112]. In predicting relapse for patients with breast cancer, Johannes et al. [177] ranked the influence of genes based on protein-protein interaction (PPI) networks and their fold-change information. This ranking was then used to dampen the chance that features are removed in Recursive Feature Elimination. Alternatively, a Bayesian approach can offer soft feature selection [178], [179]. Instead of limiting the number of significant genes, the coefficients of each gene are modeled with a Gaussian prior that depends on the covariance matrix across genes and a latent indicator for their inclusion. Insights from previous studies regarding the stability of each gene, interrelationships among genes, and gene groupings can inform the design of this prior. A similar approach has been employed in radiomic feature selection [179] and derivation of specialized lexicons from radiology reports [130], [131].

#### 2) Feature Grouping:

Prior knowledge can be used to form biologically meaningful grouping of features. Introducing such a grouping structure serves as guidance for the model to explore complex relationships among features. For instance, genes that are functionally related or belong to the same pathway or protein can be grouped together [11], [126], [173], [176]; in the preprocessing of textual data, related medical terms can be combined [154]; and nearby patches of an image can be grouped into phenotype groups as a way to model spatial heterogeneity [129]. Once groups are defined, a straightforward approach is to extract features at the group level [11], [180]. A more sophisticated strategy entails employing multi-view models to learn weights specific to features and views, as well as relationships both within and between groups.

Feature grouping has been a common technique when building models with gene expression data. While pathway-level grouping is the most common and has been shown effective in several genetic studies [173], this structure can be extended to multiple levels of hierarchy, such as genes, pathways, subfunctions, and functions. Grouping of gene expressions can be desired for several reasons. First, signature genes for the classification task are not always available across datasets due to different gene annotations across platforms, which impedes transferability. Second, gene expression profiling can be easily affected by nonbiological batch effects coming from the technical platform. Using pathways have been shown to be more reproducible and robust than treating each gene independently [180], [181]. Third, employing biologically structured inputs can facilitate model interpretation and the discovery of disease mechanisms.

#### 3) Feature Transformation:

The input features can be reformatted using scientific knowledge to more intuitively connect the inputs and outputs of the predictive task. For example, drug molecule can be represented as a graph following their chemical structure, where nodes are atoms and edges are bonds between atoms [48], [182]. Another study argued that it is not straightforward to establish connection between the physicochemical properties of drugs and the cellular mechanisms of their action [183]. Instead, the authors used associated drugs with proteins and created PPI-network based features [183].

With the growing popularity of convolutional networks like U-Net, genetic data has been transformed into “gene expression images” for training CNN models. Studies have attempted to induce meaningful spatial relationship in the transformed image by ordering gene expressions into a square matrix based on their position in a functional hierarchy [111], [112], [113], according to chromosome numbers [27], in a swim-lane organization of pathways [110], or by spectral clustering the gene expression matrix [184]. For instance, Ma et al. [111] used the hierarchical gene structure from KEGG [37] to construct a five-layer tree mapping gene expressions based on the child nodes of the tree. The tree is then converted into an image using the pivot method, considering their adjacency in the tree and sorting genes based on median values across all samples. In addition, genetic data can also be transformed into networks.

Mechanistic models can also be employed to transform input features into a more informed latent informative space, subsequently utilized for outcome prediction [98], [161]. A representative study used a mathematical model that accounts for immune system-tumor interactions to extract immunological features, which were then combined with clinical features to predict tumor size at different time points and compared with MRI results [161].

Knowledge-informed transformations of features not only enhance the predictive utility of raw data but also harmonize data collected from different platforms. Gao et al. [181] converted gene expressions into a functional spectrum of enrichment scores with respect to a public gene database so that features encode transcriptomic patterns previously demonstrated to be associated with biological function. A similar strategy can be employed for textual data. For instance, Min et al. [185] mapped clinical reports into Concept Unique Identifiers of the UMLS to extract base concepts and hierarchies of related concepts from different reports. These mappings aid in standardizing medical and non-medical terms across diverse data sources.

### C. Model Framework Design

To better adapt the natural structure inherent in knowledge, many studies proposed specialized model structures that allow incorporation of biomedical knowledge. Framework design, in this context, refers to the process of designing the modeling pipeline as well as the architecture of backbone models. Deep neural networks, in particular, provide significant flexibility to design different model architectures suitable for different forms of knowledge.

#### 1) Graph-Based Models:

Given that cancer is caused by complete processes, it is unlikely that genes act in isolation; rather, they interact with other genes through complex signaling or regulatory networks [186]. The flexibility of graph-based models has led to extensive use in genomics applications. Both the input data [48], [148], [186], [187], [188] and biological knowledge [110], [182], [189], [190] can be represented as graphs. Edges can encode relationship or similarity between biological entities, such as pathways, functional categorizations, motif gene sets, chromosomal position, and edit distance, while nodes can accommodate observed variables, such as gene expressions, as well as unobserved higher-level entities, such as proteins [148], [186]. Graph-based models provide a unique opportunity to concurrently explore multiple disease-disease, drug-drug, and drug-disease relationships. This capability enables a shift away from the traditional “one-disease, one drug” paradigm, potentially expediting drug discovery processes. Another representative work [191] built a network of nearly two thousand clinically approved drugs pooling drug-target binding profiles from multiple data sources. The proximity between two drug’s targets were measured based on the interactome between the targets of each drug [191]. Once the network was built, the topological relationship between two drug-target modules reflected whether drugs are pharmacologically distinct, complementary, similar, or independent [191].

Biological knowledge can be infused into graph-based models in several ways. Graphical models such as Graphical Convolutional Networks (GCN) can seamlessly take graphs formed from data and from knowledge as input and combine them for prediction [48], [124], [125], [126]. Edges in a graph can be pre-set based on domain knowledge or softly regularize the estimation process. In cases where a subset of edges is known to be important, but the value of their importance is unknown, Graph Attention Networks can be used to encourage the model to attend to the important edges [193].

Directed graphical models offer a distinctive advantage in their capacity for causal inference, which proves invaluable for comprehending causal relationships between biological entities. One example of this approach is a model designed to propose gene/pathway candidates that potentially enable tumor cell invasion and migration [194]. This model is based on a network with nodes representing biochemical species or phenotypes and edges denoting activations or inhibitory influences. A predefined set of logic rules determines whether a state is reachable from a node. The network was simulated as a Markov decision process to derive probabilities of each node reaching a phenotype.

#### 2) Biologically Informed Neural Network Architectures:

**Fig. 3.**
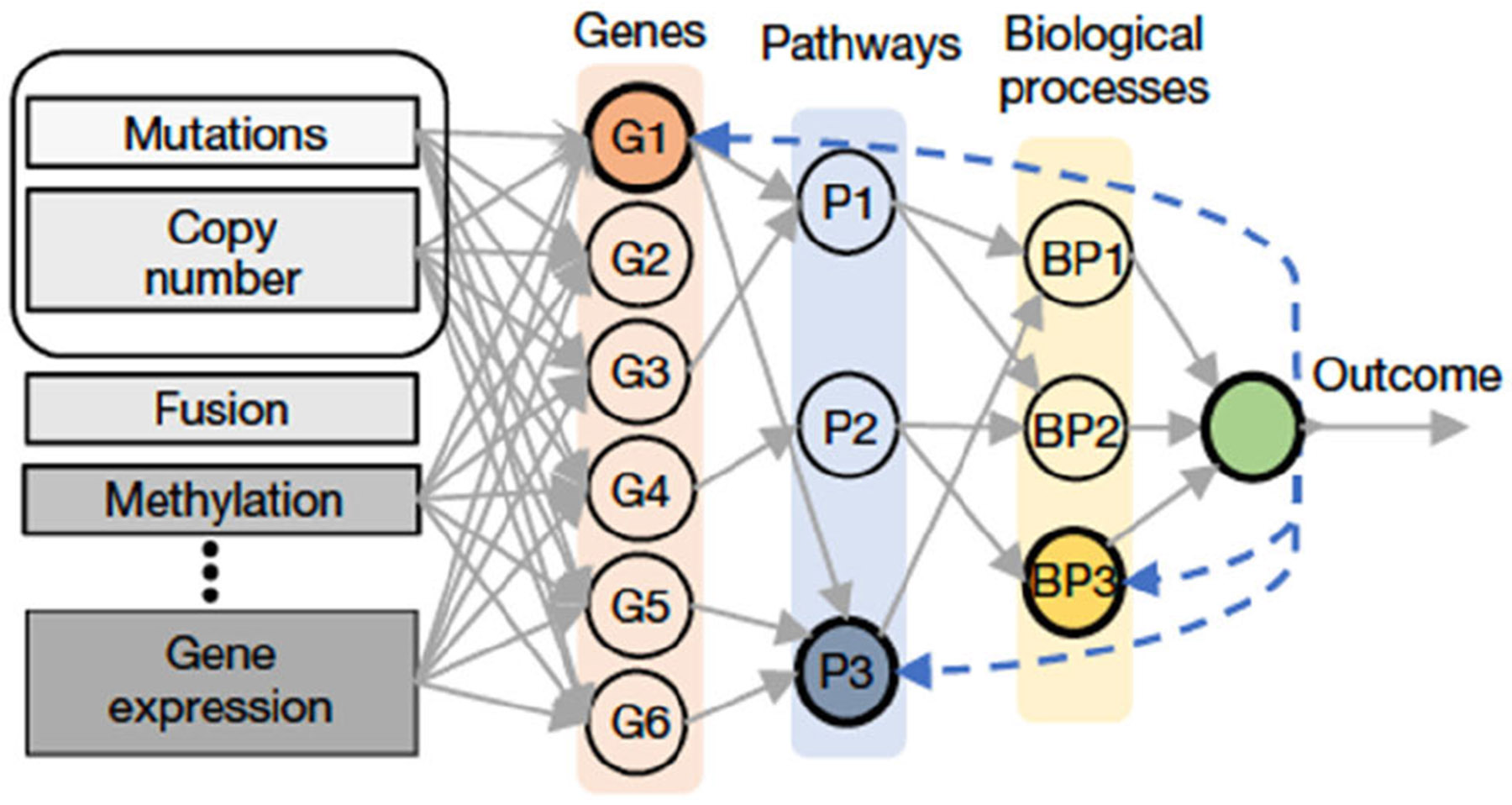
Schematic overview of the biologically-informed P-NET model.

Typically, deep neural networks are known as “black-box” machines with an extensively large number of layers and “neurons”. To improve the interpretability of these large neural networks, researchers have proposed to design customized architectures based on biological knowledge, where each layer and node have a specific biological interpretation, and only connections that follow known biological relationships are activated [94], [97], [98], [100], [102], [103], [104], [105], [106], [107]. A representative example is P-NET [195]: the input layer assimilates the molecular profile of patients as features; the second layer encapsulates a set of genes of interest chosen on the basis of domain knowledge; the subsequent five layers correspond to biological pathways and processes curated from the Reactome dataset (Figure 3). Each node in the second layer is linked to precisely three nodes in the first layer, symbolizing mutations, copy number amplifications, and copy number deletions of each gene. Connections between layers are constructed according to known parent-child relationships based on biomedical knowledge. The customized connections are implemented by multiplying a masking matrix to the weight matrix to nullify undesired connections.

Employing a biologically informed architecture can significantly improve the interpretability of black-box neural networks. Computational efficiency also improves, as the number of parameters is limited to the number of known biological relationships. However, the performance of these architectures is contingent on the quality and comprehensiveness of domain knowledge. The hard sparsity constraint also limits the network’s ability to explore unknown biological connections. One strategy involves using a hybrid architecture with both data-driven and knowledge-informed model structures. As exemplified in Seninge et al. [203], a data-driven encoder captures complex patterns from gene expression profiles, while a decoder with a single linear layer nullifies connections between the latent layer and input layer if they do not belong to a known biological abstraction. The decoder is also constrained with positive weights to maintain the explainability of the direction of biological activity.

#### 3) Customized Model Pipelines:

Apart from model architectures, the model pipeline design can also follow properties of biological relationships [121], [125], [126], [164], [194], [204], [205] or real clinical decision-making processes [122], [123], [137], [204]. As an example of mimicking the pathologist’s decision-making processes when examining WSI for breast cancer diagnosis, Mercan et al. [137] proposed a sequence of binary classification models in which a decision is made for a single diagnosis: invasive vs. non-invasive, then non-invasive samples into preinvasive and benign, and finally preinvasive samples into ductal carcinoma in situ and atypia. The underlying reasoning is that features that describe one type of diagnosis do not apply to other diagnosis.

#### 4) Customized Model Views:

The input size and receptive field are important design aspects that directly impact model’s capabilities to extract information. Input sizes, which can be modified through cropping, scaling, or patching the original input, should balance the required context and resolution needed to solve the problem. Similarly, receptive field dictates the area in input space, e.g. number of pixels in an image, that contributes to a single output unit. Domain experts can visually assess the selection of input size and receptive field as a sanity check for whether the model design suits their problem [206].

It is not expected that every part of an image contributes equally to the diagnosis, and the behavior of radiologists or pathologists when examining an image can offer valuable insights to guide the model where to “look”. A simple example is that the high-level organization of tissue at a whole-slide view is crucial for diagnosing invasive cancer, while the cellular features distinguishing preinvasive lesions are often observed at higher resolutions in a local region [137]. Various works attempted to mimic this behavior using multi-view or multi-scale DL architectures. Sha et al. [136] developed a multi-field-of-view (FOV) DL model to simulate the behavior where pathologists rely on various zoom levels when diagnosing a WSI. The model includes one main network with multiple layers for processing the large image (large FOV) and two branches with only a few layers to process small cuts of the central region of the image (small FOV). This design also ensures that the central region contributes more to the classification than the edges. Similarly, in classifying challenging nodules from ultrasound images, Liu et al. [123] pointed out that radiologists focused on the differences with surrounding tissues, as malignant nodules tend to have a blurred and irregular margin due to rapid growth. A triple-branch model was designed to mimic this behavior: the first branch analyzed low-level features, the second branch focused on context features, and the third branch extracted margin features.

Attention mechanism is another technique widely used in neural networks to encourage the model to selectively focus on certain parts of the input sequence that are most relevant to the prediction and suppress irrelevant regions. Several image-based diagnosis studies have used this technique to emphasize the suspicious or discriminative regions of the image [134], [147].

### D. Model Training

Another key component of ML is the training algorithm. The loss function is a critical component because it defines the learning direction of the ML model and quantifies how well the model performs on the given task.

#### 1) Knowledge-Regularized Objective:

Regularization can be used to incorporate knowledge into the loss function of ML models as follows:

(3) $$L_{total}=\overset{data\text{-}driven\;loss}{\overset{︷}{\sum_{i}L(f(x_{i}),y_{i})}}+\overset{knowledge\text{-}based\;loss}{\overset{︷}{\lambda \sum_{i}L_{k}(f(x_{i}),x_{i})}}$$

where $L_{k}$ measures the inconsistency with knowledge, and λ determined the relatively importance of the terms.

One approach is to drive model predictions to be consistent with knowledge such as simulation results [91], [97], probability atlas [54], [104], or clinical disease severity indicators [172] through a soft regularizer term in the objective [54], [91], [97], [104], [172] or hard constraints [53], [91], [207]. For example, Wang et al. [97] constrained the difference between the predicted tumor cell density by the proposed model and that simulated from the PI model to be small. This knowledge-infused model was shown to outperform the mechanistic model as well as data-driven models. Knowledge-based regularization can help mitigate overfitting in small sample size scenarios. A knowledge-informed label smoothing technique was introduced, where knowledge about tumor severity was distilled from clinical data and used as weak labels to regularize liver tumor segmentation models [172]. The loss function can penalize the predictive error of each sample differently. In a classification task using WSI patches [116], the loss function was modified such that a higher penalty was given when the model misclassifies patches that fall into regions that pathologists coarsely annotated as abnormal.

Another approach is to regularize feature coefficients [208], attributions [209], or alignment in the latent space [48], [210] to be consistent with prior knowledge. A Laplacian regularizer is typically used for knowledge that can be represented in a graphical form, such as protein-protein interaction networks used to encourage similar feature representation for related biological entities [210], patient similarity networks to regularize subgroups of patients to have similar predictions [49], or attribution priors to encourage similar feature contribution from functionally related genes [209]. Another commonly used regularizer is group lasso and its variants, which have been used to stimulate biologically related entities to have similar coefficients [107], [208].

Compared to the previously discussed approaches that necessitate modifying the model architecture based on prior knowledge, training models with a knowledge-regularized objective offers a more flexible method for aligning the model with existing knowledge. This approach allows for the regularization strength to be easily adjusted by modifying the values of λ. However, a drawback of this method is that it requires additional effort for hyperparameter tuning.

#### 2) Physics-Informed Neural Networks:

Physics-informed neural networks (PINNs) are neural networks that encode mathematical equations. PINNs leverage the capability of neural networks as universal approximators and use automatic differentiation to solve a customized physics-based loss. PINNs are increasingly being used to solve PDEs that are notoriously difficult to solve using standard numerical approaches. The input to the PINNs includes pairs of system state and solution values sampled at a set of locations called colocation points. Thus, PINNs are capable to train with no ground truth. If experimental data is available, an additional supervised loss term can be added as the data loss.

A representative example by [95] used PINN to efficiently solve the intravoxel incoherent motion (IVIM) model used to separate and quantify the diffusion of water molecules in diffusion-weighted imaging (DWI). IVIM have great potential for estimating prognostic cancer imaging biomarkers, however, it is rarely used clinical because of long fitting time [95]. Using a fully connected neural network of only three layers and a physics-based loss, IVIM could be solved equally or sometimes more accurately than traditional Bayesian solvers [95]. Likewise, Perez-Raya and Kandlikar [76] used PINNs to learn the parameters of the Penne’s bioheat equation, which was used to detect tumor location based on the breast surface temperature and thermal properties of the tumor. Another innovative use case of PINNs is estimating patient-specific parameters for the PI model, which additionally provided capabilities to visualize forecasts of the intermediate GBM progression curve [96].

By integrating physical laws and constraints into the training process, models are trained to adhere to fundamental principles and to respect known physical constraints. Thus, PINNs can generalize better to unseen scenarios or extrapolate beyond the training data. This is particularly useful in applications where data is limited or expensive to obtain. However, the additional computational burden of training with complex loss functions and enforcing physical constraints can be significant. In addition, PINNs often require careful tuning of hyperparameters related to the regularization terms. This can make the training process more sensitive to hyperparameter choices and may require extensive experimentation.

#### 3) Multi-Task and Meta-Learning:

Multi-task learning (MTL) models are trained to predict multiple outcomes at the same time. Studies have shown that the model’s performance on the main task can be improved via joint learning of auxiliary tasks even if these side tasks are not needed at inference time [1]. MTL is commonly used for drug response prediction as this setup allows gathering evidence from multiple drugs to find predictive genes, i.e. supervision can be shared across different tasks. If prior knowledge about task dependencies exists, such knowledge can be ejected into the model through a task relation graph [212]. For example, Ammad-ud-din et al. [176] designed a multi-view and multitask Bayesian Multiple Kernel Learning that can learn shared evidence across drugs without assuming that the same views need to be relevant to all drugs. While MTL approaches can improve efficiency by reducing the need for separate models for each task and can lead to better performance on individual tasks due to the shared knowledge, it can also introduce complications such as task interference or negative transfer, where the model’s performance on some tasks might degrade because of conflicting information from others.

Meta-learning is a generalization of multi-task learning where the model is first trained on a distribution of related tasks before being fine-tuned and evaluated on target tasks. This framework allows incorporating both auxiliary datasets and related tasks at the same time. Cho et al. [213] proposed a meta-learning approach that uses various combinations of integrated omics datasets to train a prediction model of cancer survival. Results showed that this meta-learning design allowed finding robust functional and semantic relationships among genes. Nonetheless, meta-learning requires careful design of the learning process and can be computationally intensive, as it involves training on a variety of tasks to achieve effective generalization.

#### 4) Knowledge-in-the-Loop Learning:

Experts can actively participate in the model training process by offering human-in-the-loop assessments. For instance, two experienced radiation oncologists blind-reviewed and selected preferred plans among predicted treatment plans for patients with head and neck cancer in [60]. In another study [171], a one-shot active learning approach was employed, wherein pathologists identified the most representative samples from high-fidelity samples screened by a self-distillation model. These selected samples were then labeled as clean data for noise-robust training. One-shot active learning was used where pathologists picked up the most representative samples from high-fidelity samples screened from a self-distillation model and tagged them as clean data for noise-robust training.

Domain knowledge can also be used to dynamically adjust or update model outputs during the training process. For example, Huang et al. [104] first applied a DL segmentation model to unlabeled CT scans. The segmentation outputs were compared with a probability atlas to compute a confidence map. The high confidence pixels were then selected and used as pseudo-labels for unlabeled samples. Similarly, Li et al. [103] refined their segmentation maps by warping with the probabilistic atlas map and computing the nearest interpolation.

Reinforcement Learning (RL) builds an environment where biomedical knowledge and data-driven algorithms can seamlessly interact. RL has been used in various clinical applications to control treatment doses, including cancer chemotherapy [50], [160]. In a RL framework designed to mimic cancer antiangiogenic therapy, a controller learns optimal drug administration schedules by interacting with a patient whose tumor volume is simulated via a mathematical model of tumor growth [50]. Another representative work leveraged deep reinforcement learning and multiscale PDE to simulate tumor growth, where biological knowledge played an essential role in defining the regulatory substances, diffusible factors, cancer cells, and signaling pathways as RL entities in this virtual tumor microenvironment [159].

Integrating human expertise into knowledge-in-the-loop approaches enhances model accuracy by incorporating domain-specific insights and correcting model predictions, particularly in complex domains where expert judgment is crucial. However, this approach can be resource-intensive, requiring substantial time and effort from experts, which may not be feasible for all applications. Additionally, the reliance on expert input may introduce variability and potential bias.

#### 5) Hyperparameter Setting:

Domain knowledge can also be used to set model hyperparameters. In tumor detection and segmentation models, prior knowledge about tumor size and shapes have been used to define realistic anchor boxes [122], [123]. Radiologists’ sparse annotations of prostate contour have been used as prior seed points for a ML model to produce semi-automatic segmentations [117], [118]. Bayesian models such as Bayesian Networks can utilize domain knowledge to define appropriate priors [105]. Expectations about smoothness of predicted tumor cell density maps or sparsity of predictive gene sets can also be used to determine appropriate ranges for the corresponding hyperparameters.

## V. Challenges and Future Directions

A primary limitation in developing accurate and generalizable ML models for cancer diagnosis and prognosis is the scarcity of labeled data. Despite ongoing efforts in data collection, obtaining large-scale clinical samples remains challenging and costly due to practical factors such as invasive biopsy procedures and the substantial time needed for expert annotation. Integrating biomedical knowledge with ML models serves to partially mitigate the shortage of labeled data, enhancing model effectiveness while grounding predictions in established biomedical understanding.

Another key benefit of KIML lies in improving the explainability of ‘black box machines’, particularly DL models. For real clinical usage, the ability for clinicians to understand and justify the predictions made by AI models is paramount. Explainable models provide insights into the rationale behind predictions, enabling healthcare professionals to assess the reliability and validity of these predictions in the context of patient care. More importantly, explainable models can contribute to a deeper understanding of cancer’s complex mechanisms for knowledge discovery. In contrast with existing XAI tools that allow practitioners to retrospectively validate model behavior against medical knowledge after model development, KIML approaches proactively integrate knowledge into the model training process. This proactive integration can help narrow down the hypothesis space at early stage, leading to more clinically relevant outcomes.

Moreover, the incorporation of active learning strategies, which iteratively updates the model based on successive treatment outcomes, can significantly tailor and refine the model’s performance over time. This adaptive approach is not only able to optimize sample efficiency but also ensure its improvement, aligning closely with the evolving nature of clinical treatments and patient responses. Traditional active learning involves human experts to provide feedback during the process based on their clinical assessment or experimental evaluation. This concept can be generalized as knowledge-in-the-loop learning, where the feedback system may consist of domain experts or an automated oracle constructed based on existing scientific knowledge.

Collecting large-scale public datasets for different types of tumors and drug has greatly fostered collaboration, transparency, benchmarking, and knowledge discovery in cancer diagnosis and prognosis. Public datasets facilitate the pre-training of robust models, which can be fine-tuned with smaller, specific datasets, through techniques such as transfer learning, partially mitigating the shortage of limited samples. Moreover, future research that concentrate on compiling a collective band of quantitative biomedical knowledge can greatly facilitate knowledge integration into ML models.

Several design considerations need to be carefully addressed when designing KIML models. First, there is an inherent trade-off between knowledge exploration and exploitation based on whether the modeling goal is knowledge-discovery or consistency. Different types of knowledge integration techniques are suitable for each case. For instance, biologically informed architectures constrain the model to explore relationships among known biological entities, while other techniques, such as knowledge-based regularizers, dynamically allow the model to disobey knowledge and explore new possibilities. Second, knowledge selection is not trivial, especially in unexplored problems like discovering the effect of a new drug or exploring mechanisms of unknown biological phenomenon. A systematic way to probing and selecting relevant knowledge from existing knowledge banks is needed. Third, similar to experimental data, knowledge can contain subjectivity and selection bias. Therefore, the ability to quantify uncertainty in knowledge and dynamically adjust the model’s response is desired. There is ample room for future improvements in KIML.

The integration of medical and genomic data into ML models raises significant privacy and ethical concerns that must be addressed. To protect patient data, robust measures such as anonymization, de-identification, and others are essential [214]. Additionally, the use of hybrid execution models in cloud computing environments, where sensitive data is processed within private clouds while non-sensitive data utilizes public clouds, further enhances data security [215]. These approaches are complemented by secure data storage and transmission protocols. Ethical considerations are equally vital in the responsible use of medical and genomic data. Obtaining informed consent from patients and ensuring transparency about data usage is also critical. Furthermore, compliance with regulations such as HIPAA (Health Insurance Portability and Accountability Act) and GDPR (General Data Protection Regulation) is crucial to uphold both data privacy and ethical standards. Addressing these issues is vital for safeguarding patient rights, maintaining the integrity of the research, and building trust in the use of medical and genomic data.

Scalability and generalizability are critical for the effective application of ML in cancer research. Cancer research often involves heterogeneous, high-dimensional, and multi-source datasets collected from multiple centers and different clinical trials. Scalability ensures that ML models can handle these vast and varied datasets without compromising performance. To address scalability, one of the most promising approaches is the use of distributed learning. Distributed learning enables the training of ML models on large, diverse datasets from multiple healthcare institutions without the need to centralize sensitive patient data. This is particularly important in healthcare, where data privacy and security concerns, as well as legal and ethical constraints, often prevent the sharing of data across institutions. Distributed learning techniques such as Federated Learning have been developed to tackle these challenges [216]. On the other hand, generalizability is also essential in cancer research, ensuring that findings, models, and treatments are applicable across diverse patient populations, cancer types, and clinical settings [217]. This review discussed several strategies for improving generalizability including transfer learning, which leverages knowledge from related tasks to enhance model performance on new tasks; multitask learning, where the model learns from multiple related tasks simultaneously to build shared representations; diversifying training data, which involves incorporating a wide range of data sources to ensure the model can handle various scenarios; simplifying model complexity to prevent the model from capturing noise and overfitting; and model regularization. Ensuring that a scalable model also generalizes well requires careful attention to data quality, model simplicity, cross-domain learning, and the management of biases and domain shifts. Balancing scalability with generalizability is a key challenge in developing robust and reliable ML models in cancer research.

Ultimately, the central aim is to develop user-centric AI systems that can greatly facilitate healthcare automation in advancing cancer diagnosis, prognosis, and treatment. KIML is a nonnegligible component to enhance model interpretability and reliability in building such AI systems. In the development of such systems, it is crucial to involve clinical users to provide accessible domain knowledge, define relevant model explanations, and evaluate practical utilities [128].

## VI. Conclusion

Advancements in AI have positioned ML as a powerful tool in healthcare research, capable of analyzing diverse data modalities such as radiologic images, electronic health records, histopathologic slides, and molecular profiles. Integrating biomedical knowledge into ML models offers a promising direction to address the modeling challenges in cancer prognosis and diagnosis. This review underscores the KIML in enhancing model performance, generalizability, and interpretability by incorporating biomedical knowledge into classic and deep learning models. We detail key strategies for effective knowledge representation and integration across various data types. Despite the wealth of biomedical knowledge available, considerable opportunities for further exploration exist. Continued collaboration between ML scientists and medical experts is essential for developing sophisticated, knowledge-informed analytical tools that can advance cancer diagnostics and prognostics.

## Figures and Tables

**TABLE I:** Summary of Main Cancer Datasets With Their Capabilities (c) and Limitations (l)

Type of data	Description	Capabilities	Limitations
Clinical	Contain a spectrum of patient-related information, including demographics, medical history, laboratory test results, symptom logs, performance status, questionnaires, health records, and more	(C1) Diverse perspectives of patients’ health condition (C2) Relatively inexpensive to collect and is often collected as part of the standard clinical routine	(L1) Unstructured and heterogeneous (L2) Limited standardization standards across centers (L3) Textual data require models that can handle sequential data, thus not straightforward to fuse with other data modalities
Radiologic Imaging	Medical examinations done through imaging technologies such as Magnetic Resonance Imaging (MRI), Positron Emission Tomography (PET), Computer Tomographic (CT), X-rays, ultrasound, and mammography	(C1) Non-invasive (C2) Provide high-level morphologic and phenotypic measurements about the tumor and its surrounding tissue (C3) Existence of commonly used standardization and preprocessing protocols	(L1) Acquisition is expensive: requires specialized equipment and each imaging modality comes with additional acquisition cost (L2) Image resolution and noise level depend on the imaging technology (L3) Presence of artifacts (L4) Inter-observer variability in the diagnosis
Pathologic Imaging	Image taken from tissue samples obtained from patients through biopsy procedure	(C1) Provide low-level characterization of cell-level features (C2) Offers definitive discriminant power of a true diagnosis finding abnormalities at molecular level	(L1) Require invasive tissue collection (L2) Constraints in data collection such as tissue sample location and quantity (L3) Time-consuming labeling and shortage of pathologists with expertise to annotate datasets (L4) Inter-observer variability in the diagnosis (L5) Large size whole-slide images that demand more memory and computational resources
Molecular	Describe the abundance or status of molecules in tissue samples. Multi-omics data span genomics, epigenomics, proteomics, transcriptomics, etc. Molecular interactions data describe potential function of molecules through their interactions with other partners.	(C1) Provide low-level characterization of cell-level features (C2) Multiple assays (multi-omics) can be conducted for each tissue sample (C3) Provide insights into tumor heterogeneity and progression (C4) Data can be collected from single cell experiments instead of biopsy	(L1) Expensive to acquire (L2) Expensive to conduct multi-omic assays (L3) Constraints in data collection such as tissue sample location and quantity (L4) High-dimensional outputs with high sparsity, measurement variability, and noise
Treatment	Information about cancer treatment plans such as radiotherapy or chemotherapy doses and medication usage.	(C1) Important for monitoring post-treatment tumor progression (C2) Relatively cheap to collect; generally already available	(L1) Generally reported as mean dose; spatial distribution of doses across the target tissue/organ is not always available

**TABLE II:** Summary of Forms of Knowledge Representation and Their (C), Limitations (L), and Integration Strategies (M)

Representation	Description	Capabilities and Limitations	Integration strategy
Scientific Knowledge			
Mathematical Models	Knowledge expressed through mathematical expressions that contain variables or constants. Examples: PT model of glioblastomas[70], model of immune system-tumor interactions[72]	(C1) Well-defined and mathematically-expressed (C2) Interpretability and transparency (C3) Generalization for being extensively validated through scientific experiments (L1) Limited expressiveness and personalization, may not capture more complex relationships (L2) Computationally expensive and slow to run if the equation has no closed form solution (L3) Limited availability	(M1) Simulation as training data [23], [50], [86], [97], [98], [146], [159], [160], [161] (M2) Knowledge-regularized objective [86], [91], [92], [93], [94], [95], [97], [99], [100] (M3) Physics-informed neural networks [76], [95], [96] (M4) Feature transformation [98], [161] (M5) Knowledge-in-the-loop learning [50], [159], [160]
Probabilistic Relations	Knowledge expressed as connections between variables in a probabilistic or stochastic setting. Examples: gene mutation rates, correlations, probability distributions	(C1) Contains uncertainty quantification of the knowledge so can be more robust to noisy data (C2) Easy to incorporate as prior in Bayesian models (L1) Imprecise and weak form of supervision	(M1) Feature transformation [101] (M2) Knowledge-in-the-loop learning [103],[104] (M3) Knowledge-regularized objective [102],[208] (M4) Hyperparameter setting [105]
Knowledge Graphs	Knowledge expressed as a graph where vertices represent concepts or biological entities and edges denote relationships between them. Examples: gene databases, proteinprotein interaction networks	(C1) Structured representation facilitates explainability of complex relationships (C2) Several large-scale knowledge-graphs are publicly accessible (L1) Gaps in knowledge may limit model performance (L2) Struggles to integrate with unstructured data	(M1) Knowledge-regularized objective [107], [195], [207], [208], [209] (M2) Biologically-informed neural network architectures [126], [162], [186], [194]-[202] (M3) Feature selection, grouping, and transformation [52], [108]-[111], [172], [175][177], [180]-[183], [188], [189] (M4) Customized model pipelines [193] (M5) Graph-based models [48], [187], [190], [191], [192]
Knowledge from experts			
Quantitative knowledge from experts	Quantitative assessments provided by experts based on their medical experience. Examples: tumor location annotations, treatment plan selections, lesion size estimates	(C1) Direct feedback relevant to the target task (L1) Require time-consuming, case by case assessments by medical experts (L2) Inter-observer variability	(M1) Weakly supervised learning [32], [114], [115], [116], [120], [125], [164], [170] (M2) Customized model pipelines [121],[122] (M3) Hyperparameter setting [117],[118],[122] (M4) Knowledge-in-the-loop learning [60],[171] (M5) Feature selection and transformation[51], [55], [56], [63], [155], [178]
Qualitative knowledge from experts	Non-numerical information derived from observations, subjective experiences, and behaviors of experts from their clinical practice. Examples: glaze of pathologists when examining an image, “rules of thumb” in diagnosis	(C1) Information often imprecise (C2) Generally available at low acquisition costs (L1) Not straightforward to express mathematically or to incorporate into the model. (L2) Not well-defined and subjective (L3) Imprecise supervision	(M1) Feature selection and transformation [124], [129], [130], [131], [135], [149] (M2) Customized model pipelines [123], [125], [137], [204] (M3) Customized model views [134], [136] (M4) Knowledge-regularized objective [35], [175]
Auxiliary datasets			
Public datasets	Openly accessible and shared collections of data, typically collected from large-scale studies and from multiple centers, spanning various formats such as image, text, omics, dictionaries. Examples: TCGA, PubMed, UMLS, KEGG, ImageNet	(C1) Wide accessibility (C2) Available with large number of samples, modalities, and different disease conditions (C3) Facilitates transparent benchmarking (C4) Covers diverse topics and applications (L1) Data quality variability (L2) Not tailored to specific research questions (L3) Overused or outdated data	(M1) Transfer learning [22], [109], [130], [144], [145], [156], [157], [165] (M2) Weakly supervised learning [167], [168] (M3) Feature selection, grouping, transformations [11], [132], [146], [184] (M4) Customized model pipelines [203] (M5) Multi-task/meta-learning [153], [212] (M6) Graph-based models [185]
Private datasets	Datasets that access is restricted to authorized individuals or entities to protect sensitive information and comply with legal standards. Examples: pre-clinical data, patients with other types of cancer	(C1) Exclusive access to proprietary information to foster unique insights and discoveries (C2) Control over data quality (C3) Tailored to specific research questions (L1) High acquisition cost (L2) Limited collaboration (L3) Challenges in benchmarking	(M1) Transfer learning [135], [165]-[169] (M2) Knowledge-regularized objective [48] (M3) Feature transformation [53], [140], [145]
